# Editorial: Psychological implications of preterm birth

**DOI:** 10.3389/fpsyg.2025.1572801

**Published:** 2025-02-28

**Authors:** Livio Provenzi, Minesh Khashu, Mia McLean, Evalotte Morelius

**Affiliations:** ^1^Department of Brain and Behavioral Neuroscience, University of Pavia, Pavia, Italy; ^2^Developmental Psychobiology Lab, IRCCS Mondino Foundation, Pavia, Italy; ^3^Neonatal Service, University Hospitals Dorset, Poole, United Kingdom; ^4^Department of Psychology and Neuroscience, Auckland University of Technology, Auckland, New Zealand; ^5^Department of Health, Medicine and Caring Sciences, Linköping University, Linköping, Sweden; ^6^School of Nursing and Midwifery, Edith Cowan University, Joondalup, WA, Australia; ^7^School of Nursing, University of Jordan, Amman, Jordan

**Keywords:** preterm birth, stress, parenting, Neonatal Intensive Care Unit (NICU), developmental care, nurturing care

## Too early. And then?

Preterm birth, which occurs in approximately 10% of births worldwide (Ohuma et al., 2023), has far-reaching implications for the child, parents, and extended family members. While the physical health challenges associated with preterm birth are extensively documented (Camerota et al., 2023; Crump, 2020), the psychological repercussions—both short- and long-term—remain partially underexplored. This gap in knowledge is concerning, as preterm birth not only presents significant physical and mental health burdens but also imposes substantial economic costs on society (Frey and Klebanoff, 2016). Understanding the psychological implications of preterm birth is essential for developing effective interventions and support systems for affected individuals and families.

The experience of preterm birth and the subsequent hospitalization in the Neonatal Intensive Care Unit (NICU) can be extraordinarily distressing for both infants (Cong et al., 2017) and parents (Caporali et al., 2020). In the NICU, the infant is often exposed to an environment filled with overwhelming sensory stimuli, such as bright lights (Aita et al., 2013), loud noises (Casavant et al., 2017), and frequent medical procedures (Carbajal et al., 2008). These stressors, compounded by the painful separation from their parents, may have a profound impact on the infant's socio-emotional and cognitive development (McLean et al., 2022a), partly through epigenetic modifications of genes critical for stress regulation (Provenzi et al., 2015, 2020). Additionally, the psychological strain on parents during this critical period cannot be understated. The uncertainty surrounding their infant's health, coupled with the stress of navigating the NICU environment, can lead to significant mental health challenges for parents, including anxiety, depression, and post-traumatic stress disorder (PTSD) (Axelin et al., 2022; McLean et al., 2022b; Persson et al., 2023).

Moreover, the psychological effects of early interventions, both during and after the NICU stay, are a critical area of concern. Since medical advancements have improved the survival rates of preterm infants, the focus of NICU professionals needs to shift beyond survival, to quality of life and optimizing long term health outcomes through family-centered care programs (Aita and Snider, 2003; Alberts et al., 2024). Yet, the potential long-term psychological consequences of early medical care and hospitalizations are not fully understood. As infants transition from the NICU to home and beyond, the quality of the parent-child relationship emerges as one of the most important protective factors in supporting healthy developmental trajectories (Provenzi et al., 2015; Toole et al., 2024). Research suggests that early parent-infant interactions, from the NICU to the early years of life, play a crucial role in promoting emotional and cognitive resilience in preterm infants (Arwehed et al., 2024; Spence et al., 2023). However, the influence of these interactions on long-term psychological outcomes warrants further investigation.

This Research Topic seeks to address these important gaps in knowledge, bringing together interdisciplinary perspectives on the psychological implications of preterm birth for both infants and parents. We aim to explore the complex interplay between early medical experiences, parental wellbeing, and child development, with a focus on the psychological mechanisms at play. By advancing our understanding in this area, we hope to contribute to the development of targeted interventions that can mitigate the psychological challenges faced by preterm infants and their families, ultimately improving their quality of life and long-term outcomes.

## What can you find in this article collection?

The present Research Topic features contribution from interdisciplinary groups coming from many different countries: from New Zealand to Ukraine, from Spain to China, from France to US, from Italy to Germany, from Switzerland to Finland. The contributions can be clustered into three main areas: psychological outcomes of preterm birth and NICU stay in preterm-born individuals and their parents; promoting early interventions for preterm infants and their parents matters; innovative approaches to study the psychological implications of preterm birth.

### Psychological outcomes of preterm birth and NICU stay in preterm-born individuals and their parents

Lee et al. assessed executive functioning (EF) in adolescents born very preterm (VPT, < 32 weeks) at age 17. The results showed that VPT adolescents performed worse than full-term (FT) peers in areas such as working memory, planning, and cognitive flexibility, with the most significant deficits seen in those born before 28 weeks. The relationship between gestational age and EF was mediated by neonatal medical complexity and white matter abnormalities. These findings emphasize the need for continued cognitive support, especially for those with higher medical and neurological risks.

Another contribution (Pavlyshyn et al.) explored the connection between stress markers in preterm infants during their NICU stay and developmental outcomes at 24–30 months. The study found positive correlations between melatonin levels and communication and problem-solving skills, while cortisol levels were negatively correlated with these abilities. Longer NICU stays and mechanical ventilation were also predictive of developmental delays. This highlights the critical impact of neonatal stress on later development and the importance of addressing medical factors during early life.

In the study by Martinez-Shaw et al., the health-related quality of life (HRQoL) of 8-year-old children born preterm with very low birth weight (VLBW) was examined. The study revealed that VLBW children reported better HRQoL than the general population, with maternal stress and social support acting as key mediators between perinatal factors and child HRQoL. This underscores the importance of considering maternal wellbeing in interventions aimed at improving the quality of life for families with VLBW children.

Jean-Dit-Pannel et al. focused on the emotional and psychological challenges fathers face when navigating the complexities of having a preterm infant in the NICU during the COVID-19 pandemic. COVID-19 restrictions led to separations, affecting fathers' perceived paternal identity. Additionally, concerns about the infant's development and COVID-19 health risks heightened fathers' vulnerability to postpartum depression. This highlights the compounded psychological stress fathers experience during this period, underlining the need for targeted mental health support.

Huang et al. conducted a study on the needs of grandparents of preterm infants in the NICU and how demographic factors influence these needs. The study identified that grandparents primarily sought reassurance regarding the quality of care, followed by information and proximity to the infant. Addressing these needs is crucial for alleviating the emotional burden on families, particularly grandparents, of preterm infants.

### Promoting early interventions for preterm infants and their parents matters

Filippa et al. studied the effects of maternal singing and speaking on the general movements (GMs) of preterm infants in the NICU. The intervention group engaged in maternal vocalizations three times per week for 2 weeks, and GMs were assessed at term-equivalent and 3 months corrected age. Results showed significant improvements in GMs for the intervention group. Maternal vocal interaction could enhance neurobehavioral development in preterm infants and should be integrated into NICU care routines.

Ludwig et al. present the results from two randomized controlled trials compared standard care (SC) with SC plus Family Nurture Intervention (FNI) in level-4 NICUs. The FNI program focused on mother-infant interactions to foster autonomic emotional connection. The trials demonstrated significant long-term benefits for infant neurobehavior, development, and the autonomic health of both mother and child. These findings support the integration of FNI into preterm care and provide a new perspective on emotional co-regulation between mother and infant.

Trautmann-Villalba et al. explored the impact of skin-to-skin contact (SSC) on emotional and behavioral outcomes in children born preterm. The study, part of the Deisy Study, assessed 33 children aged 6–8 years and found that parental stress 6 months post-discharge was the main predictor of emotional and behavioral issues. However, SSC immediately after birth did not significantly influence these outcomes. This highlights the importance of addressing parental stress in the care of preterm infants.

In another review of the literature, Leppänen et al. revised psychosocial parent-infant interventions for preterm infants, analyzing 22 studies published from 2000 to 2024. Most interventions focused on counseling and emotional support, and the majority showed positive effects on the parent-child relationship. The findings emphasize the need for more standardized, long-term research to better support families with preterm infants, particularly those at higher risk.

### Innovative approaches to study the psychological implications of preterm birth

In the paper by Billeci et al. a study protocol investigating the impact of early video-feedback (VF) intervention on brain-to-brain co-regulation between very preterm (VPT) infants and their mothers is anticipated. The study uses EEG hyperscanning to assess brain synchronization during mother-infant interactions, comparing VPT and full-term (FT) dyads. VPT dyads will receive the VF intervention post-discharge, and outcomes such as mother-infant interaction and maternal mental state will be measured at 3, 6, and 9 months. The study aims to enhance understanding of VF interventions and their impact on infant development and maternal wellbeing.

Sadjadpour et al. compared the performance of logistic regression with machine learning (ML) models in identifying parents at risk for depression after their infant's NICU admission. Data from 300 parents of NICU infants was analyzed using eight ML algorithms to predict depression risk. Results showed that all models, including logistic regression, had high performance in identifying at-risk parents. Logistic regression provided a reliable tool for targeted depression screening in NICU parents.

## Conclusion

In summary, this Research Topic highlights the importance of considering both the medical and psychological aspects of preterm birth and not just for the infant but for the whole family and larger society and healthcare policy makers and providers. To improve the quality of life and long-term outcomes for preterm-born children and their families, it is crucial to integrate emotional support with medical care. By advancing our understanding of these complex dynamics, we can develop more effective and comprehensive care strategies for this vulnerable population.
